# Epigenetics and ADHD: Reflections on Current Knowledge, Research Priorities and Translational Potential

**DOI:** 10.1007/s40291-022-00609-y

**Published:** 2022-08-06

**Authors:** Charlotte A. M. Cecil, Joel T. Nigg

**Affiliations:** 1 Department of Child and Adolescent Psychiatry/Psychology, Erasmus MC-Sophia, Rotterdam, The Netherlands; 2 Department of Epidemiology, Erasmus MC, Rotterdam, The Netherlands; 3 Molecular Epidemiology, Department of Biomedical Data Sciences, Leiden University Medical Center, Leiden, The Netherlands; 4 Division of Psychology, Department of Psychiatry, Oregon Health and Science University, Portland, OR, USA

## Abstract

Attention-deficit/hyperactivity disorder (ADHD) is a common and debilitating neurodevelopmental disorder influenced by both genetic and environmental factors, typically identified in the school-age years but hypothesized to have developmental origins beginning in utero. To improve current strategies for prediction, prevention and treatment, a central challenge is to delineate how, at a molecular level, genetic and environmental influences jointly shape ADHD risk, phenotypic presentation, and developmental course. Epigenetic processes that regulate gene expression, such as DNA methylation, have emerged as a promising molecular system in the search for both biomarkers and mechanisms to address this challenge. In this *Current Opinion*, we discuss the relevance of epigenetics (specifically DNA methylation) for ADHD research and clinical practice, starting with the current state of knowledge, what challenges we have yet to overcome, and what the future may hold in terms of methylation-based applications for personalized medicine in ADHD. We conclude that the field of epigenetics and ADHD is promising but is still in its infancy, and the potential for transformative translational applications remains a distant goal. Nevertheless, rapid methodological advances, together with the rise of collaborative science and increased availability of high-quality, longitudinal data make this a thriving research area that in future may contribute to the development of new tools for improved prediction, management, and treatment of ADHD.

## 1 Introduction

Mental disorders, including psychiatric, neurodevelopmental and substance use disorders, collectively comprise the single greatest source of health burden in the world [1]. Problems with inattention, disorganization, impulsivity, and hyperactivity are a surprisingly important factor. When severe and impairing, these problems are usefully organized clinically into a syndrome called attention-deficit/hyperactivity disorder (ADHD). ADHD and other mental disorders are thought to emerge from a complex and developmentally dynamic interplay of genetic and environmental influences, beginning as early as fetal life [2]. Epigenetic processes that modulate gene expression have been proposed as a key biological marker (and potentially a molecular mediator) of genetic and environmental influences on ADHD. In this *Current Opinion* piece, we discuss the status and promise of epigenetic studies for ADHD research and clinical practice. We focus specifically on DNA methylation (DNAm), as it is, to date, the most widely examined epigenetic marker in mental disorders, including ADHD. We begin by providing a background of ADHD and its complex etiology, how epigenetics may fit in the mix, and what lessons we have learned from epigenetic research on ADHD. From there, we outline key knowledge gaps and challenges going forward, laying out a roadmap for future research priorities in this area. We conclude with an optimistic, but critical view on possible translational applications that may emerge from the use of methylation-based profiling towards improved prediction, detection, stratification, and treatment of ADHD.

### 1.1 Attention-Deficit/Hyperactivity Disorder: Core Features and Current Scientific and Clinical Challenges

At first, ADHD may seem a minor player in the saga of mental health-related impairments, alongside such crippling conditions as major depression, severe alcoholism, bipolar disorder, and schizophrenia. Indeed, some children under the ADHD umbrella do attain a relatively benign outcome with manageable remaining difficulties, and when stimulant medications work, as they do in a majority of cases, the short-term benefit is among the most dramatic in psychiatry. However, a closer look reveals ADHD to in fact be vastly underappreciated in the matrix of mental health-related burden and cost [3].

ADHD is one of the most prevalent neurodevelopmental disorders worldwide, affecting 3–5% of the general pediatric population when defined stringently, with only minimal regional variations in prevalence [4, 5]. It has solid psychometric validity with regard to its statistical structure as well as the reliability and validity of diagnostic assignment [6]. More commonly diagnosed in males during childhood, ADHD is primarily characterized by inattentive, impulsive, and hyperactive behavior that manifests along a continuum in the population (with ADHD diagnosis representing the tail end of a dimensional syndrome) [3]. ADHD emerges earlier than most other psychiatric disorders, typically onsetting in the preschool years, peaking around mid-childhood, and in a substantial proportion of cases, persisting over time, with an estimated prevalence of 2.5% in adulthood [7]. Consistent with its early age of onset and association with subtle changes in neural structure and neural network functional architecture, ADHD can be hypothesized to have roots in early (fetal) neurodevelopment [6, 8], although its pathophysiology remains largely unknown.

ADHD, like other mental disorders, is diagnosed behaviorally, without a biological test. To qualify for the condition, children must have excessive levels of inattention (which includes features of disorganization such as losing their things) and/or hyperactivity/impulsivity. These levels of behavioral expression must be (1) not explained by defiance or failure to understand; (2) not better explained by another disorder; (3) extreme for developmental stage and/or age; (4) persistent (at least 6 months); (5) present in at least two settings (e.g., home and school); and (6) clearly interfere with social, academic, or occupational functioning [9].

Challenges to accurate diagnosis include the fact that inattention, impulsivity, and hyperactivity are normal childhood behaviors, therefore evaluating their extremity requires sophisticated knowledge of child development as well as careful evaluation. Another challenge is that the symptoms are not always specific; for example, inattention can be due to anxiety, depression, learning problems, fatigue, or physical illness, while impulsivity can be due to anxiety or fatigue. Furthermore, it is often accompanied by other features such as learning problems, irritable mood and tantrums, and motor or speech delays that can confound diagnosis. Thus, expert clinical knowledge of associated conditions is also necessary. A final challenge is that, like many medical and psychiatric conditions, ADHD can present in different ways at different times. Children (or adults) can appear primarily inattentive or primarily hyperactive, or both [9]. The diagnosis has remained controversial, at least in the US, in part due to steadily rising rates of case identification [10], possibly due to rising rates of identification of milder cases [11]. Despite all these challenges, when carefully evaluated by knowledgeable clinicians adhering to formal diagnostic criteria, ADHD is one of the most reliable diagnoses in the nosology [6].

At the same time, evidence suggests that, to a large extent, ADHD symptoms behave like a trait in the population, at least in terms of its genetic architecture [12]. Thus, biological research on ADHD has to include careful expert evaluation of caseness. This is a clear challenge in very large sample studies when detailed evaluation is cost prohibitive. Fortunately, standardized research tools can overcome this challenge to an extent that appears to be adequate for most purposes. When they are not, the option exists to treat ADHD as a behavioral dimension, for which reliable measurement is much more readily established and cost effective. While it remains possible that extreme symptoms of ADHD may have unique determinants (e.g., unusual environmental exposures or genetic mutations), we treat ADHD and dimensional symptoms of ADHD as synonymous in this paper for ease of presentation.

We highlight that a striking feature of ADHD is its extensive relationship to other mental disorders: as well as co-occurring with other neurodevelopmental, behavioral, and emotional problems in childhood (e.g. autism spectrum dis-order, conduct disorder, anxiety disorders, and later, emerging mood disorders), ADHD substantially increases the risk for secondary mental health problems in adolescence and adulthood, such as substance abuse, depression, and psychosis [6]. It appears to be part of a causal pathway toward these other problems, making it an important focus for primary and secondary prevention [13, 14]. Crucially, from a human and public health perspective, ADHD is associated with serious negative life outcomes, including accidental injury, suicide, lower educational attainment, unemployment, poor physical health, and premature mortality [6, 15]. Taken together, these features make ADHD a central target for understanding and addressing the growing burden of mental disorders in the population.

Despite continued improvements in the characterization of ADHD and its neurobiological correlates over the past three decades [16, 17], observed effect sizes for biological signals (whether genetic, neuroimaging, or other) are too small, and generally do not apply to all children with ADHD, for clinical utility at this juncture [3]. As a result, and perhaps unsurprisingly given its protean presentations and diverse influences, the use of biomarkers to enhance personalized medicine in ADHD has achieved only limited progress and has yet to have a substantial impact on clinical care. Scientific advances in biomarker development are particularly needed in three key domains.

The first is *early risk detection*. If ADHD risk could be known with sufficient confidence in very early life, when development is most malleable, then early subtle disturbances of neural or behavioral development should be more readily rescued and potential low-cost preventive care could be more routinely provided. Well-accepted examples from other fields include gestational diabetes, where risk is routinely identified and low-risk prevention undertaken, as well as nursing problems after birth, which are routinely addressed with behavioral/parenting guidance. Perhaps analogous procedures would be possible for neurodevelopmental concerns if they could be identified early enough, enabling the timely delivery of interventions. As it stands however, known risk factors for ADHD show poor specificity and weak predictive utility. Furthermore, early detection still relies almost exclusively on the presence of ADHD symptoms once they have already developed, often too late for the implementation of low-cost/low-risk intervention [6]. The development of new (molecular) risk prediction tools, ideally presymptom manifestation, could open the door to new ways of detecting risk earlier and better, helping to identify individuals at greatest need of support and moving us one step closer to primary prevention. In this respect, the potential to identify risk prenatally is particularly powerful.

The second domain concerns secondary prevention once ADHD is in place, which requires advances in *patient stratification and subtyping*. Individuals with ADHD show substantial heterogeneity in terms of phenotypic presentation, presence of comorbidities, developmental course, and stability (i.e. with symptoms remitting for some children, while becoming chronic for others). Despite substantial recent progress in characterizing this heterogeneity [17], neuro-biological mechanisms or biomarkers of sufficient power to enhance care still remain to be clarified. The identification of potential biomarkers (which do not need to be mechanistic to be useful) related to heterogeneity, and thus to course, would be an important advance and appears increasingly realistic.

The third domain concerns *treatment formulation*. While currently available treatments (e.g. stimulant medications) are effective in many cases, an estimated 30% of individuals do not respond to these treatments, and even in those who do, long-term outcomes often remain disappointing and concerning [18–20]. Here, new tools are needed to help clinicians better predict treatment response and formulate more personalized treatment plans, which could in turn improve prognosis. Ultimately, the discovery of actionable causal mechanisms underlying ADHD may also lead to the identification of new pharmaceutical, nutraceutical, or behavioral interventions with greater efficacy.

In what follows, we discuss whether and how epigenetic markers may in future help us to address these challenges. However, before proceeding, readers should be aware of criticisms and controversies that, historically, have attended biological research on ADHD. First, the extensive use of pharmacological intervention in children has led to long-standing concerns about overmedicalization, particularly in the US. Second, the association of ADHD with poor social adjustment and aggression has fueled concern about over-biologizing of psychosocially rooted problems (and risk of racial bias in biological research given associations of psychosocial risk with race and ethnicity). Third, and more recently, interest in supporting variation in ‘neurotypical’ development has provoked controversy about overpathologizing of putative personality variations that may be socially difficult but not truly a disorder. While the philosophical roots of these issues are longstanding and too extensive to be addressed herein, and attention to such concerns should not deter rapid progress in understanding within-person developmental mechanisms and biomarkers, awareness of these social issues facilitates contextualizing what follows in relation to the eventual need for placing future findings in broader ethical and community context.

### 1.2 Context for Epigenetic Studies: What Do We Know About the Etiology of ADHD?

**Fig. 1:**
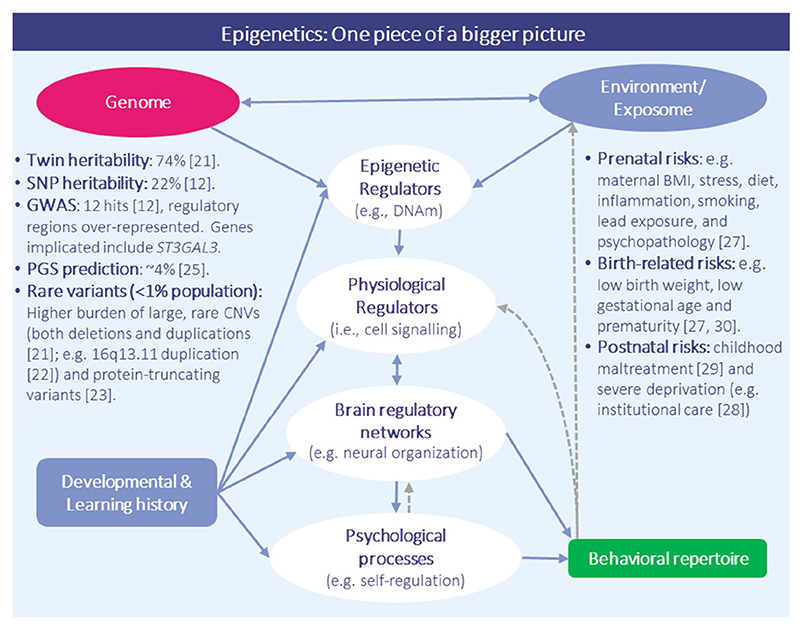
ADHD as a complex, multifactorial entity involving interactions across environmental, biological, and behavioral domains. According to current theoretical models of ADHD, such as the ‘mechanistic-cybernetic model’ [2], multiple extrinsic (e.g., environmental factors, blue), intrinsic (genetic, pink; biological and psychological processes, white) and behavioral (green) domains are thought to interact together to shape risk and resilience in ADHD development. Key findings to date from research on both genetic (left-hand side) and environmental (right-hand side) influences on ADHD are highlighted. While this *Current Opinion* focuses specifically on the potential relevance of epigenetics in ADHD research and clinical practice, it is important to note that epigenetic processes are likely only one of several interconnected domains implicated in the patho-physiology of ADHD. Note: double-headed blue arrows = bidirectional influence; single-headed blue arrow = directional influence; grey dotted arrow = correlated but not causal association. *ADHD* attention-deficit/hyperactivity disorder, *SNP* single nucleotide polymorphism, *GWAS* genome-wide association study, *DNAm* DNA methylation, *BMI* body mass index, *PGS* polygenic score

The field has strong consensus that both genetic (G) and environmental (E) factors influence the emergence of ADHD [6] (for an overview of key findings to date, see Fig.1). Heritability estimates for ADHD are substantial (twin-based estimates: circa 74% [21]; estimates based on single nucleotide polymorphisms [SNPs]: circa 22% [12]), reflecting a polygenic genetic liability involving many common genetic polymorphisms of small effect [12] as well as rarer genetic mutations of larger effect in a minority of cases (e.g. with a higher burden of rare, large copy number variant [CNV] deletions and duplications [22] as well as protein-truncating variants [23] observed in individuals with ADHD). Of interest, genetic liability for ADHD diagnosis correlates highly with that of (1) continuous/subclinical ADHD symptoms, supporting ADHD as a heritable *dimensional* trait in most instances; and (2) other child- and adult-onset psychiatric disorders, further positioning ADHD within a liability pathway to poor mental health [12, 24]. However, to date, the predictive ability of polygenic risk scores for ADHD is in itself too low to be clinically useful (circa 4% variance explained in broadly defined ADHD based on a recent meta-analysis [25]), highlighting the large gap that still exists between our knowledge of (and ability to model) genetic effects on ADHD versus what is to be expected based on heritability estimates [26]. For more information on the genetics of ADHD, we refer the reader to several excellent reviews on the topic (e.g. [21, 24, 26]).

At the same time, ADHD has been clearly linked with numerous environmental risk factors, particularly around the prenatal and perinatal period. Some of the most robust risk factors identified are maternal prenatal health conditions and psychological distress (e.g. hypertension, obesity, pre-eclampsia, immune activation), in utero exposure to poor diet (with critical factors still being determined), teratogenic effects of certain medications (e.g. acetaminophen) and environmental exposures (e.g. lead), as well as neonatal factors such as prematurity and low birth weight [27]. Other extreme exposures in the postnatal environment (such as extreme infant emotional neglect) have also been associated with an ADHD syndrome [28, 29]. Yet, to what extent these factors are causal remains, in most cases, an open question. Recently, studies employing advanced causal inference methods have helped to better separate associations due to confounding versus likely causal drivers. For example, studies have shown that for factors such as maternal smoking, effects appear to reflect mainly genetic or familial confounding, while for others, such as birth weight, effects are supported by causally informative designs [27, 30]. However, even when causal effects are supported, risk factors tend to show small effects and be associated with many other outcomes besides ADHD.

So how exactly does ADHD emerge from what is in most cases a plethora of non-specific, common factors of small effect? The answer to this question likely rests with genotype-environment interplay across development (see Fig.1). A plausible scenario is that beginning in fetal life (and perhaps even before that), environmental exposures coact dynamically with genetic liabilities over time to shape ADHD risk, phenotypic presentation, and developmental course. Nigg et al. [3] presented a model of staged emergence beginning with particular temperament manifestations in early life. In line with these models and the fetal origin hypothesis, a large study from the iPSYCH consortium recently showed that many prenatal and birth-related risk factors are associated with genetic liability for ADHD, pointing to substantial G-E overlap, with both additive (i.e. G+E) and interactive (i.e. G×E) effects on ADHD observed [31]. Whether and how this statistical interplay occurs at a molecular level is however much less clear. In recent years, epigenetic processes that regulate gene expression, such as DNAm, have emerged as both a potential biological marker and mediator of this interplay on ADHD.

### 1.3 DNA Methylation: A Potential Epigenetic Marker of G-E Interplay, Health Status and Disease Risk

Epigenetic processes regulate when (in time) and where (in the body) genes are expressed. In this *Current Opinion*, we focus specifically on DNA methylation (DNAm), as it is currently the best understood and most widely examined epigenetic marker in relation to ADHD. It is important to note, however, that DNAm operates in concert with other epigenetic mechanisms (e.g. chromatin remodeling, histone modifications, microRNAs) that are likely to also be relevant for the study of ADHD, representing an important area for future research. DNAm can tag, stabilize or control regulation of genomic regions via the addition of methyl molecules to DNA base pairs, typically in the context of cytosine-guanine (CpG) dinucleotides.[32]. Although developmentally dynamic and potentially reversible, DNAm patterns are typically mitotically passed on during cell division, which can lead to long-term changes in gene activity and downstream phenotypes [33]. Growing interest in DNAm stems from evidence that (1) it is influenced by both genetic and environmental factors as early as pregnancy (e.g. dietary, chemical, psychosocial exposures [34, 35]; (2) it plays an essential role in normative development, including brain maturation and function [36]; and (3) disruptions in DNAm patterns associate with numerous health outcomes, including neurodevelopmental and psychiatric disorders [37, 38].

These properties, along with the ease with which they can be evaluated in peripheral tissues, make DNAm an attractive molecular system in the search for both novel biomarkers and disease mechanisms. For example, aberrations in DNAm appear to play a causal role in certain disorders (e.g. genomic imprinting disorders [39]), and to mediate the effect of specific genetic and environmental influences on health outcomes (e.g. the effect of genetic mutations on cancer development [40] or the effect of tobacco smoking on lung function [41]). Furthermore, recent studies have shown that DNAm patterns are best explained by the joint (i.e. additive and interactive) contribution of genetic and pre/postnatal environmental factors, as opposed to either factor in isolation [42, 43], pointing to DNAm as a candidate mechanism for gene-environment interplay across development; however, cautions are in order. The promise of epigenetics as a mechanism for disease risk has at times been overstated in popularized format. While we do argue for great promise here, limitations and challenges are very real. One major limiting factor for mechanistic research in humans concerns the cell-type and tissue-specific nature of DNAm patterns. This is especially problematic for the study of psychiatric phenotypes such as ADHD, as DNAm patterns measured in easily accessible tissues (e.g. blood, saliva) may not reflect those in the (relatively inaccessible) organ of interest, i.e. the central nervous system (CNS). In fact, although a sizable minority of DNAm sites show strong cross-tissue correspondence (with some sites showing stronger variation across individuals than tissues), most DNAm varies substantially between peripheral and brain tissues (and even between different brain cells and regions [34, 44]). While postmortem studies can help address this challenge by measuring DNAm directly in the brain, they come with their own limitations, including the use of small samples with mixed clinical presentation and incomplete phenotyping, limited data on relevant brain regions, and the reliance on mainly adult samples, which may not generalize to neurodevelopmental conditions such as ADHD in children [45].

While between-tissue and cell-type variation in DNAm will render mechanistic discovery slow, it does not undercut the potential of DNAm as a biological marker for disease prediction, stratification, and diagnosis. This application is especially well-suited for use in peripheral tissues, which are more readily available *in vivo* even if it may not be causal for the phenotype of interest. For example, certain exposures (e.g. early life stress) may leave system-wide signatures, even though the primary mechanistic effect on the phenotype is neural [46]. Based on peripheral DNAm patterns alone, it is already possible to estimate a range of exposures, traits, and health outcomes using algorithms trained from large datasets (e.g. age, smoking, BMI [47]), and to detect certain diseases sooner and more accurately than conventional diagnostic methods, leading to improved clinical care [48–50]. Although these developments have already led to important breakthroughs across multiple fields (e.g. epidemiology [51], clinical genetics [37], forensics [52], ageing [53], oncology [50]), methylation-based profiling has yet to sub-stantively impact mental health research and practice. This lag, at least in part, reflects the specific challenges within the field of psychiatric epigenetics, including the characteristics of psychiatric phenotypes per se (i.e. their clinical heterogeneity, low specificity and ‘fuzzy’ diagnostic boundaries, making them particularly difficult targets to study), as well as the more limited availability of sufficiently powered datasets (e.g. compared with phenotypes that are more readily defined and routinely assessed, such as BMI, age, or smoking status). Nevertheless, interesting and even surprising insights are beginning to emerge from epigenetic research on ADHD, thanks to rapid methodological advances and the establishment of large-scale collaborative efforts. These are discussed below.

## 2 Epigenetic Research on ADHD to Date: Key Findings and Lessons Learned

### 2.1 Cross-Sectional Case-Control Studies: *VIPR2* Methylation as the Most Consistent Finding

Early epigenetic studies on ADHD focused on specific candidate genes (e.g. dopaminergic genes) within relatively small samples, yielding mixed findings (for a review, see [38]). In time, the increased availability and decreased cost of methylation arrays has led to a shift towards the use of hypothesis-free approaches (following in the footsteps of genetics), by allowing researchers to interrogate hundreds of thousands of DNAm sites across the genome in relation to ADHD. As shown in Table 1, most of these epigenome-wide association studies (EWASs) have been performed comparing peripheral DNAm patterns between ADHD cases versus controls (in children [54–56], adolescents [57], and adults [58]). While only one of these studies detected genome-wide significant differences in DNAm between groups [58] (likely due to limited power in available datasets), some promising targets have been identified, with the most notable example being *VIPR2* methylation.

*VIPR2* encodes a receptor for vasoactive intestinal peptide, a small neuropeptide that is widely expressed in the CNS where it functions as both a neurotransmitter and neuroendocrine hormone, regulating several processes relevant for mood and behavior such as circadian rhythm [54]. Methylation of this gene has been implicated in ADHD by multiple clinical studies, including one study in a sample of boys aged 7-12 years [54], a second study in a predominantly male sample of monozygotic twin pairs discordant for the disorder (mean age 10 years [55]), and most recently in the largest clinical EWAS to date comparing 391 children with clinically established ADHD versus 213 nonpsychiatric controls (age range 7-12 years [56]). Interestingly, the study by Mooney et al. [56], which included both males and females, found that this association is sex-dependent: whereas boys with ADHD showed lower *VIPR2* methylation, girls with ADHD showed higher methylation relative to controls.

This finding highlights the potential value of considering sex interactions on DNAm, especially for phenotypes that show strong sex differences such as ADHD, something that is rarely done in current research. The existence of sex-dependent effects may also explain why *VIPR2* associations have not been replicated in the only two other case-control EWASs performed to date, as those analyses included both males and females but were not stratified by sex [57, 58]. Providing further support for a role of *VIRP2* in ADHD is evidence from independent studies reporting associations between *VIRP2* methylation and several known risk factors for ADHD, including prepregnancy maternal overweight [59], prenatal tobacco smoking [60] and early nutrition [61], with the latter study identifying *VIRP2* methylation as a locus linking early malnutrition with later deficits in cognitive function and attention. Together, this evidence points to *VIRP2* as an interesting candidate for future research, and as a potential marker of early environmental influences on (sex-specific) ADHD risk.

Besides *VIRP2*, other DNAm loci have also been identified within single clinical EWASs but these await independent replication (e.g. suggestive sites annotated to *SLC7A8* and *MARK2* in the study by Mooney et al. [56]; genome-wide significant differences in sites annotated to *PCNXL3*, *DENND2D*, *PWWP2B* and *UBASH3A* in the study by Rovira et al. [58]). Furthermore, as analyses have been based on case-control comparisons, it is unclear to what extent the identified DNAm patterns associate with disease severity. At a broader level, several of these EWASs have found that ADHD-associated DNAm patterns are enriched for biological pathways involved in inflammatory processes, fatty acid oxidation, and neurotransmitter function [54, 55, 57]. However, these findings are hard to compare directly between studies and will necessitate further work to better understand their potential functional relevance.

Although DNAm differences in *VIPR2* emerge as a somewhat consistent finding, it is rather sobering to see that generally there is little convergence in the specific DNAm sites identified by the case-control EWASs performed to date. Aside from small samples (for what turn out to be small effects of individual DNAm sites) and differences in the type of peripheral tissue examined (e.g. saliva vs. blood), a key reason for mixed findings may be the use of crosssectional designs (i.e. with DNAm and ADHD symptoms assessed at the same time) in samples of varying age and developmental stage, which raise two important issues. First, these designs leave it unclear whether the identified DNAm patterns represent an antecedent (e.g. reflecting genetic or environmental risk factors for ADHD), a mere correlate (e.g. due to smoking or other behaviors associated with ADHD) or a consequence (e.g. as a result of medication use or as part of the disease process itself) of ADHD. Disentangling the direction of these associations is necessary for informing which methylation-based applications may be most suitable for use in ADHD research and clinical practice. Second, the analysis of samples varying in age is challenging due to the highly dynamic and developmentally dependent nature of DNAm. This was recently illustrated by a large-scale study characterizing epigenome-wide changes in DNAm over the first two decades of life, which found that over half of DNAm sites change significantly with time, and can do so in a non-linear way (i.e. with DNAm levels changing at different rates across development [62]). This raises the possibility that DNAm patterns contributing to ADHD susceptibility may also differ across time [63].

### 2.2 Prospective Population-Based Studies: Evidence of Epigenetic Timing Effects on ADHD Symptoms

While still exceedingly rare, the increased availability of population-based cohorts with repeated epigenetic data, in which the same individuals are followed *longitudinally* starting from birth, is opening unprecedented opportunities to address many of these issues, by enabling us to map the relationship between DNAm and ADHD as it unfolds over time. In particular, these cohorts can begin to clarify (1) whether DNAm levels measured at birth, i.e. *before* symptom onset, associate with the development of ADHD later on; and (2) whether these associations remain stable or change across time. Results to date have been intriguing (see Table 1).

The first population-based EWAS of ADHD was performed by Walton et al. [64] using data from over 800 children from the Avon Longitudinal Study of Parents and Children (ALSPAC [65]). Interestingly, the authors found that DNAm patterns at birth differed between children who went on to follow a chronic high versus low ADHD symptom trajectory from age 7–15 years; however, this signal was no longer detectable when using DNAm at age 7 years. Specifically, of the 13 genome-wide significant DNAm hits identified at birth, none met corrected significance thresholds when measured again at age 7 years. The top hits identified at birth were annotated to genes involved in multiple processes, including neurodevelopment and fatty acid oxidation (which has also been implicated in several of the clinical studies as discussed above [54, 57]). Strikingly, one of these hits (cg09989037) was annotated to *ST3GAL3*, a gene that 2 years later was discovered as the strongest *genetic* (as opposed to epigenetic) locus associated with ADHD in the largest genome-wide association study (GWAS) to date [12]. This suggests that the neonatal epigenetic signal detected by Walton et al. may in part reflect (and potentially mediate) genetic liability for ADHD, an hypothesis supported by recent evidence of a functional relationship between genetic and epigenetic variation at this specific locus [66], although environmental influences are also at play [34]. *ST3GAL3* encodes an enzyme involved in the sialylation of glycoproteins, an essential process for brain development and neurotransmission. In humans, mutations in *ST3GAL3* have been linked to a range of phenotypes relevant to ADHD (e.g. developmental delays, cognitive and motor impairments [67, 68]), and inactivation of this gene in animals results in marked cognitive deficits, reduced motor coordination and hyperactivity [69, 70], effects that seem to be largely mediated by disrupted brain myelination, a known neural correlate of ADHD [71].

**Fig. 2:**
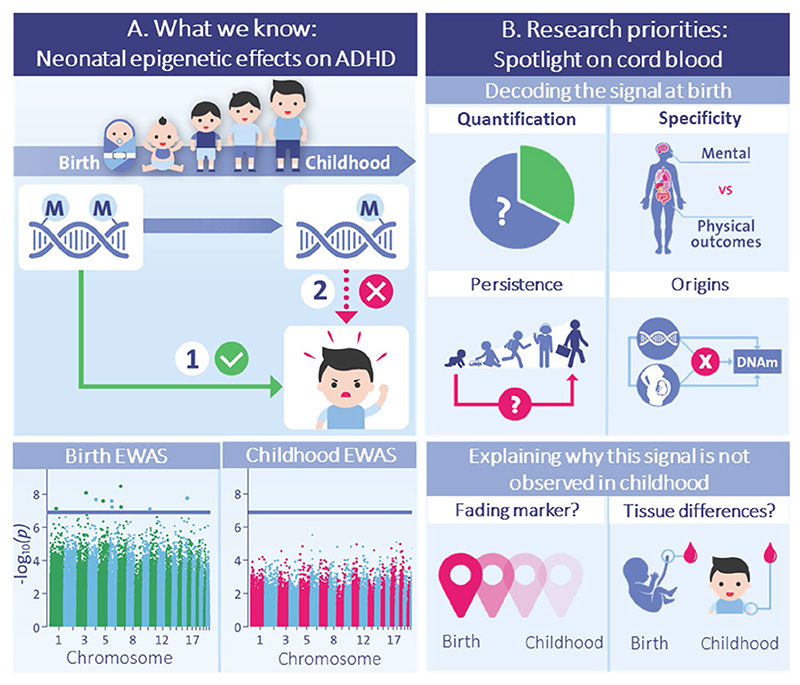
Key gap 1: Explaining the neonatal epigenetic signal for ADHD risk. **a** Top panel: General pattern of epigenetic timing effects observed by EWAS studies in population-based birth cohorts [64, 72], indicating that (1) DNAm patterns at birth prospectively associate with ADHD symptoms in childhood; and (2) DNAm patterns in childhood do not associate cross-sectionally with ADHD symptoms in childhood. Bottom panel: EWAS Manhattan plots from the meta-analysis by Neumann et al. [72] showing that differences in signal are evident both in terms of the identification of prospective, but not cross-sectional, genome-wide significant associations (i.e., ‘dots’ above the blue genome-wide correction line), as well as the overall association ‘signal’ detected across the genome at birth versus in childhood (i.e., height of the bars in the Manhattan plot). **b** Top panel: Future research will be needed to characterize key properties of the epigenetic signal detected at birth, in order to evaluate its potential as a possible biomarker for early risk detection, *presymptom manifestation*, including (1) how much variance in ADHD it explains (*quantification*); (2) to what extent this signal is specific to ADHD compared with other child mental and physical outcomes (*specificity*); (3) whether this signal at birth continues to predict ADHD and related outcomes in adulthood (*persistence*); and (4) what genetic and environmental influences drive this signal in the first place (*origins*). Bottom panel: In future, studies will also need to clarify why the epigenetic signal identified at birth is no longer detected from DNAm in childhood, for example due to ‘fading’ predictive power (e.g. DNAm at birth may tag genetic or prenatal influences on ADHD, with this signal becoming noisier in time due to the accumulation of postnatal influences on DNAm) or tissue and cell-type differences between time points (e.g., with cord blood containing unique types of multipotent cells that disappear rapidly after birth, potentially explaining why the signal at birth is not detected in peripheral blood later in life). *ADHD* attention-deficit/hyperactivity disorder, *EWAS* epigenome-wide association studies, *DNAm* DNA methylation

While intriguing, the findings from Walton et al. were based on a single cohort, making their reliability and generalizability difficult to establish. Recently, Neumann et al. [72] re-examined the question of epigenetic timing effects on ADHD in a meta-analysis of nearly 2500 school-aged children from multiple cohorts participating in the Pregnancy and Childhood Epigenetics (PACE) consortium [73], including the dataset from Walton et al. Remarkably, the same general pattern of results was confirmed: DNAm patterns at birth associated with ADHD symptoms in childhood (i.e. prospective EWAS meta-analysis), but DNAm patterns in childhood did not (i.e. cross-sectional EWAS meta-analysis) (see Fig. 2a). This difference in signal was not just explained by the inclusion of ALSPAC data (i.e. the same cohort used by Walton et al.) or by differences in sample size across time points. Hits at birth included a DNAm site located in the promoter region of *ERC2*, a gene highly expressed in the brain that plays a role in neurotransmitter release and has been associated with cognitive functioning [74], as well as a DNAm site in the *CREB5* gene, which is involved in neurite outgrowth and has been previously implicated in ADHD diagnosis by genetic studies [75]. Of note, the specific sites identified by Walton et al. were not significantly associated with ADHD symptoms in this meta-analysis (although eight sites, including the one annotated to *ST3GAL3*, showed a consistent direction of associations), which may be explained by differences in how ADHD symptoms were modeled (i.e. trajectory-based comparisons capturing a more severe phenotype versus dimensional analyses). However, both studies do converge in showing that neonatal epigenetic patterns carry more signal as a predictor of ADHD risk than those measured later in childhood.

### 2.3 Why is the Potential Existence of Epigenetic Timing Effects on ADHD Meaningful?

The finding that neonatal prediction of DNAm to ADHD symptoms would be stronger than in childhood is at first look counter-intuitive. Typically, two variables that are measured close in time tend to be more strongly associated than variables measured years apart. Aside from underscoring the dynamic nature of DNAm/ADHD associations, this finding has two major implications: (1) it supports the potential of DNAm as an early biological marker of ADHD risk, presymptom manifestation; and (2) it suggests that to benefit from this potential marker, the timing of assessment could be crucial, i.e. the particular DNAm risk signal captured at birth may no longer be detectable when DNAm is measured later in life. The same principle may also hold for other developmental/life stages, although this remains to be established. More broadly, variability in epigenetic prediction over time may help to explain inconsistent findings in the literature, as most studies have sampled DNAm at widely varying ages. They may also explain why the only other population-based study of ADHD to date, a cross-sectional EWAS of over 4500 adults, failed to identify significant associations to ADHD symptoms [76]. Above all, the identification of a neonatal risk signal could inform the development of a much-needed new predictive tool for improved early detection, via methylation-based profiling. If developmental staging is involved [3], it is also possible that these neonatal DNAm markers may correspond to related, mediating traits more so than to ADHD by childhood.

## 3 Key Gaps: Defining a Roadmap of Research Priorities for ADHD Epigenetic Research in Humans

Taken together, current findings lay a solid foundation for future studies by (1) pinpointing specific targets for further investigation (e.g. *VIPR2*, *ST3GAL3*); (2) highlighting the need to consider sex-specific effects; and (3) underscoring the importance of considering developmental timing and age effects, with cord blood emerging as one particularly promising source of signal for ADHD risk prediction, pre-symptom manifestation. However, important gaps need to be addressed before we can realistically evaluate the utility of DNAm as a molecular tool for personalized medicine in ADHD.

Doing so will require three major considerations. First, from a biomarker perspective, we will need to shift our focus from individual DNAm sites of small biological effect, towards the identification and use of broader poly-epigenetic signatures, following in the footsteps of psychiatric genetics. Second, it will be necessary to adopt a more developmentally sensitive, life-course approach to the study of epigenetics and ADHD, in order to map dynamic associations over time. Third, strong collaboration and integration of research will be essential across both samples (e.g. population-based and clinical studies) and disciplines (e.g. bridging the biological and psychological sciences; human observational data and in vivo/in vitro experimental models; bench-to-bedside research), in order to identify robust biomarkers and move towards novel mechanistic insights. In response to these needs, several large-scale initiatives have already emerged. These include TEMPO, a new European-led project aiming to shed light on epigenetic timing effects on ADHD and the potential utility of neonatal DNAm as a predictive tool, as well as the recent creation of an ADHD-Epigenetics Working Group within the Psychiatric Genetics Consortium (PGC), dedicated to advancing capabilities and methods for longitudinal modeling of DNAm/ADHD associations across the life-course. In this section, we highlight some of the key questions that need to be addressed to help move the field forward.

### 3.1 Key Gap 1: Decoding the Epigenetic Signal at Birth

As reviewed above, DNAm patterns at birth appear to associate more strongly with ADHD symptoms than DNAm patterns measured later in childhood. As a next step, we will need to better understand this neonatal signal in order to evaluate its potential as a tool for early risk prediction, by answering the following questions (also visualized in Fig. 2b, top panel)

#### 3.1.1 How Much Variance in ADHD Does this Neonatal Signal Actually Explain?

To date, evidence of neonatal effects has been based on EWAS studies, which examine how single DNAm sites along the genome associate with ADHD, with each site typically showing small effects. However, a central question in the field is how much variance in ADHD is *jointly* explained by *genome-wide* DNAm variation. To address this question, future studies could apply methods recently adapted from genetics, such as aggregate poly-epigenetic risk scores (comparable with PRS [77]) or methods that rely on genome-wide similarity matrices (comparable with genome-wide complex trait analysis [GCTA] [78]; for epigenetic data, see [79, 80]), although the latter require access to very large datasets. Once this overall signal has been quantified, it will then be possible to compare it with that of other known risk factors for ADHD (e.g., PRS scores, prenatal risks, gestational age, and birth weight), and test whether it adds unique predictive power over and above these factors. It will also be important to examine whether neonatal DNAm explains variance in clinically relevant ADHD (e.g., diagnosis), as to date it has only been associated with (subclinical) symptoms in the general population.

#### 3.1.2 How Specific is this Signal to ADHD?

Second, it will be valuable to map the ‘phenotypic boundaries’ of this neonatal signal to establish its specificity to ADHD as a trait or disorder, versus associated neurodevelopmental and health outcomes. This is crucial because use of neonatal DNAm as a predictive tool for early risk detection would require defining the boundaries of what is being predicted. Interestingly, other recent meta-analyses from the PACE consortium (using largely overlapping datasets as those in Neumann et al. [72]) did not mirror the ADHD finding that epigenetic patterns at birth were associated with other mental or physical health outcomes more strongly than epigenetic patterns measured later in childhood (e.g., cognition [81], general psychopathology [82], BMI [83]), supporting a degree of specificity. On the other hand, there is preliminary evidence that, similarly to what was observed for ADHD, peripheral DNAm patterns at birth associate more strongly with trajectories of social communication deficits (one of the hallmarks of ASD) than DNAm patterns at later time points [84], although findings remain to be confirmed in a multi-cohort setting. This may suggest that neonatal DNAm patterns may be useful for detecting risk for multiple neurodevelopmental conditions, but not for general psychopathology. In future, a more systematic approach will be needed in order to compare neonatal signals across a larger range of developmental phenotypes, for example using multi-phenotype approaches adapted from genetics [85].

#### 3.1.3 How Far into Development Does the Neonatal Signal Predict?

Evidence to date links DNAm patterns at birth with ADHD symptoms measured in childhood [64, 72]; however, the lack of longitudinal studies spanning several decades has precluded the possibility of testing whether these associations extend into the adolescent or even adult years. This question is important because by adulthood, the burden of (untreated) ADHD increases, affecting functional and societal outcomes such as employment and productivity, healthcare utilization, parenting and relationship quality, which makes the search for early predictors of chronicity even more relevant. Currently, participants in some of the longest-running epigenetic birth cohorts are reaching their 20s and early 30s, enabling for the first time to test whether DNAm patterns at birth associate with ADHD in adulthood, and conversely, the extent to which adult ADHD is connected to biological variation very early in life. In the meantime, cohorts tracking change from childhood into adolescence are likely to continue to yield findings that will ultimately have to be reconciled with those from the infant cohorts. Findings will be informative no matter what: if an association from early life to adolescence or adulthood holds up, it means that DNAm patterns at birth may help to identify individuals who are more likely to show a persistent trajectory of symptoms and who may thus benefit the most from the implementation of early strategies to curb risk. Conversely, if prediction by neonatal DNAm stops beyond childhood, this would indicate that while still potentially useful as an early marker of ADHD risk, other signals may be more informative for tracking stability and persistence of symptoms over time.

#### 3.1.4 What Factors Drive the Neonatal Signal?

The last question relates to why we find an epigenetic signal at birth to begin with: could it be capturing (and potentially propagating) the effect of specific prenatal environmental (e.g., maternal inflammation or stress), developmental (e.g., biological age), or genetic (e.g., common or rare mutations) influences on ADHD? Answering this question is fundamental to evaluate whether DNAm at birth may have utility as an *exposure* biomarker to assess the presence of early risk factors for ADHD. Of note, while many studies support a role of both genetic and environmental influences on DNAm, these factors continue to be typically examined separately. This is problematic for two reasons. First, it means that environmental exposures associated with DNAm patterns may be largely genetically confounded, and as such not likely to be effective intervention targets. Second, genetic effects on DNAm may in turn be environmentally modulated (e.g., genetic nurturance), and as such potentially actionable [86]. As such, integrated, genetically informed epigenetic analyses will become increasingly important. For example, family-based studies could be used to *separate* G-E effects, by testing whether discordance in prenatal exposures also associates with discordance in this epigenetic signal at birth and downstream ADHD symptoms, independently of genetic influences. At the same time, molecular genetic data in large-scale cohorts may be leveraged to model *joint* G-E effects, which would instead allow us to identify specific genetic variants and prenatal exposures that additively or interactively influence DNAm patterns associated with ADHD [42, 87].

### 3.2 Key Gap 2: Tracking Dynamic DNAm/ADHD Associations Over the Life Course

Aside from better characterizing the role of neonatal DNAm in ADHD risk, another key priority will be the use of longitudinal data to track temporal change and potential bidirectional associations between DNAm and ADHD symptoms from early life to adulthood. This will be necessary to tackle the following outstanding questions.

#### 3.2.1 Why do Epigenetic Associations Apparently Differ between Birth and Childhood?

Several scenarios may explain observed epigenetic timing effects, as illustrated in Fig. 2b (bottom panel). On the one hand, it is possible that DNAm patterns at birth act as a better *proxy of ‘risk load’* for ADHD compared with DNAm patterns later in childhood. Indeed, considering that many risk factors for ADHD (e.g. genetic liability, prenatal exposures, perinatal factors) are already present before or around birth, it is plausible that they may be captured most strongly by neonatal epigenetic patterns. Indeed, some prenatal risk factors for ADHD (e.g., exposure to tobacco smoking) may be more accurately measured using DNAm at birth even compared with alternative methods such as self-report, which can be more vulnerable to measurement error (e.g., due to recall bias, non-disclosure, etc. [88]). As time passes, this epigenetic signal may become ‘noisier’ due to the accumulation of postnatal influences on DNAm (e.g., environmental and immune changes), and thus no longer detectable when DNAm is measured at later time points (i.e., fading marker).

On the other hand, it is also possible that what is being picked up in this set of findings is not timing effects but *tissue- and cell-type composition* changes between these time periods. To date, longitudinal studies on ADHD have relied on two sources of bulk tissue: (1) DNAm at birth derived from umbilical cord blood, and (2) DNAm in childhood from peripheral blood (venipuncture) or from saliva. While the first two are both ‘blood’ (presumably offspring blood, although that is not always clear from the cord blood reports), these tissue types differ in important ways, with cord blood having a very distinct immune profile [89] as well as containing additional types of cells (e.g., multipotent cells) that rapidly decline from circulation after birth [90]. Of note, current EWASs are not optimally able to account for these differences between tissues, as the available algorithms used to correct for cell-type proportions do not estimate the presence of cells uniquely found in cord blood (except for nucleated red blood cells [91]). This is especially problematic given that cord blood is typically used as a ‘baseline’ measure for later epigenetic measurements, but we cannot tell for certain whether observed changes may be due to developmental or tissue-related differences. In future, the generation of new epigenetic data from multiple paired tissues and cell-types (including those that are present specifically around birth vs. abundant across development) will be necessary to disentangle their role in epigenetic timing effects, create more complete reference panels to adjust for cell-type composition, and establish their relative utility as predictors of ADHD.

#### 3.2.2 Do Other Epigenetic Signals Emerge After ADHD Onset?

**Fig. 3:**
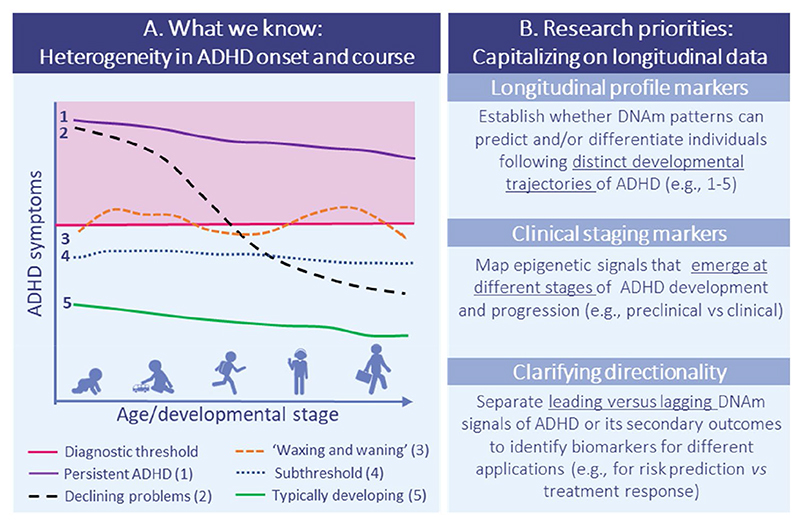
Key gap 2: Tracking dynamic DNAm/ADHD associations over the life course. (**a**) Epigenome-wide association studies. Examples of different longitudinal profiles of ADHD based on what has been reported in the literature, illustrating the large individual heterogeneity in the onset, level, and persistence of ADHD symptoms from early life to adulthood. Of note, there is ongoing debate about the existence of an ‘adult-onset’ ADHD trajectory, which necessitates more research going forward. Furthermore, while it is clear from existing research that longitudinal profiles of ADHD are heterogeneous across individuals, the specific developmental pathways remain hypothetical. (**b**) Research priorities for future research making use of repeated measures of both DNAm and ADHD symptoms. *DNAm* DNA methylation, *ADHD* attention-deficit/hyperactivity disorder

Besides clarifying observed differences between signals at birth versus childhood, the emerging use of longitudinal datasets with repeatedly assessed DNAm will be essential to move past the current reliance on cross-sectional designs, and to establish whether other epigenetic signals may emerge at different stages of ADHD development and progression (Fig. 3). This will also be critical for biomarker evaluation to determine which biomarkers are leading versus lagging indicators of ADHD or its secondary complications or outcomes. Additionally, epigenetic patterns might provide meaningful information about specific features of ADHD, helping to address individual heterogeneity in longitudinal profiles of ADHD, phenotypic presentation or subdimensions (hyperactivity, impulsivity, or associated problems such as irritability or cognitive problems), severity (e.g. transition from subclinical symptoms to clinical diagnosis), comorbidity, and treatment response, which could in future be used to guide risk stratification and clinical decision making.

Separating these potential signals however will be no small feat. The large volume of epigenetic data collected at single time points (with commonly used methylation arrays yielding DNAm levels for around 450–800k sites across the genome, a number that is likely to increase further in future) already poses challenges for psychiatric epigenetic research, especially given the relatively small samples available (with the largest clinical study to date including 329 children with ADHD [56], and the largest population-based pediatric studies including a little over 1000 individuals per time point, with well under 200 ADHD cases in those datasets [72, 73]). This limits statistical power to detect individual DNAm site effects due to the heavy multiple testing burden, and suggests that in the near term, use of methods to reduce this multiple testing burden will be essential (e.g. aggregate polyepigenetic scores [47, 92], region-level association analyses [93], and network-based analyses [94]).

While studies featuring repeated measures of DNAm and ADHD are still rare, they will become increasingly commonplace, as interest in psychiatric epigenetics continues to rise and the cost of epigenotyping decreases. Consequently, the need for new approaches capable of handling longitudinal epigenome-wide data will also grow. To date, the two published longitudinal EWASs on ADHD both modeled repeated measures of the outcome (trajectory-based analyses [64] vs. linear mixed models [72]) but not the DNAm data (i.e., separate EWASs were performed using DNAm at birth vs. in childhood). Elsewhere, however, linear mixed models have been successfully implemented to examine longitudinal change in DNAm across development [62], and methods originating from different fields, such as epidemiology and psychometrics, have also been applied to repeated DNAm data (e.g., structured life-course modeling analysis [95]; structural equation modeling [96]). In future, it will be interesting to see whether strategies that are currently used to reduce the dimensionality of epigenetic data at single time points (e.g., polyepigenetic scores and network-based analyses) could be extended longitudinally to track dynamic associations between DNAm and ADHD over time. Furthermore, time-course integration approaches developed for use with other omics data types (e.g., gene expression, metabolomics [97]) may enable model longitudinal changes in DNAm in relation to ADHD, while also making full use of the high-dimensionality of the data. Of note, recent work mapping normative epigenome-wide trajectories of DNAm across development, spanning birth to age 18 years [62], could be leveraged to characterize whether DNAm sites implicated in ADHD risk show similar developmental trajectories, and, importantly, what environmental and/or genetic factors drive change within these sites. Alongside these variable-centered approaches, person-centered approaches (e.g., latent profile analysis or community detection analyses) could also be applied to test whether individuals with different ADHD symptom profiles also differ in their longitudinal epigenetic trajectories, or whether those with different epigenetic profiles differ in outcome. Finally, accompanying the question of clinical and diagnostic prediction is the question of treatment response. Analysis of DNAm pre-versus post-treatment within clinical studies will be critical for establishing whether response to different treatments can be predicted or discerned based on DNAm profiles.

### 3.3 Key Gap 3: Mechanistic Insights into the Link Between Peripheral DNAm, Brain, and ADHD

**Fig. 4:**
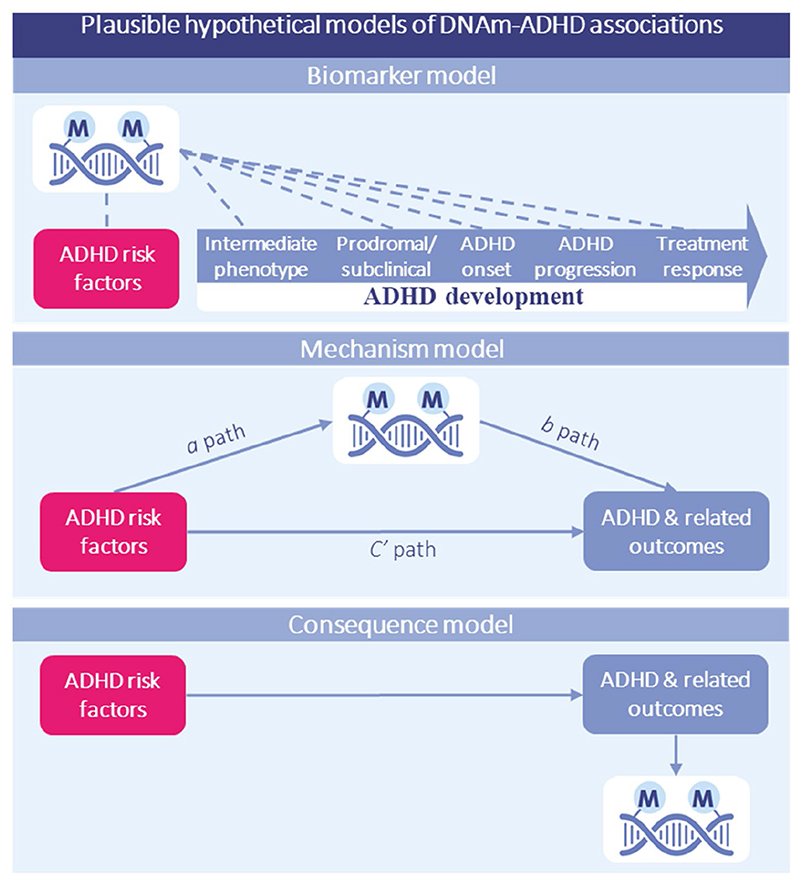
Key gap 3: Mechanistic insights into the link between peripheral DNAm and ADHD. Plausible hypothetical models explaining observed DNAm/ADHD associations. Note that these models are not mutually exclusive (e.g., DNAm alterations may be a causal factor in, as well as a consequence of, ADHD) and other models are also possible, which may emerge from future research (e.g., DNAm may function as a moderator of genetic and environmental influences on outcomes). Top panel: The (non-causal) biomarker model, whereby DNAm may tag multiple aspects relevant to ADHD, including associated risk factors (e.g. genetic liability or environmental risks such as maternal prenatal inflammation; *exposure biomarker*), intermediate phenotypes (e.g. neonatal developmental status, motor functioning or brain features; *risk prediction biomarker*), subclinical symptoms (e.g. hyperactivity and impulsivity symptoms; *early detection biomarker*), disorder onset (e.g. ADHD diagnosis; *diagnostic biomarker*), progression (e.g. change in ADHD levels over time, remittance; *prognostic biomarker*) and treatment response (e.g. stimulant medication or psychosocial intervention; *treatment response*). This application may be especially well-suited for use in peripheral tissues, which are more readily available in vivo but may not be causal for the phenotype of interest, and effective when DNAm is assessed as an aggregate polyepigenetic score (capturing broader DNAm ‘signatures’) in combination with other markers as part of a multimodal assessment tool. Middle panel: The (simplified) causal ‘mechanism’ model, whereby risk factors (independent variable) are hypothesized to partly influence ADHD and related outcomes (dependent variable) via changes in DNAm (mediator variable). Bottom panel: The (simplified) causal ‘consequence’ model, whereby DNAm patterns may be altered by ADHD and related factors, for example as a consequence of medication use (e.g., psychostimulant medication) or the disease process itself. An important priority for future research will be to test these different models by using more advanced causal inference approaches (e.g., experimental animal and in vitro designs), as well as to characterize how peripheral DNAm patterns associated with ADHD relate to the brain itself—the most relevant organ for ADHD and other mental disorders. *DNAm* DNA methylation, *ADHD* attention-deficit/hyperactivity disorder

As our ability to detect epigenetic signals associated with ADHD at different stages of disorder onset and progression increases (e.g., via collaborative, longitudinal initiatives), so will the need to move past correlational designs towards causally informative designs. DNAm marks can be useful as prediction and etiology biomarkers even in the absence of causal effects; however, they are far less likely to guide intervention targets unless they lie on a causal or mechanistic pathway to ADHD, or outcomes. A schematic overview of the possible role of DNAm as a (non-causal) ADHD biomarker, mechanism or consequence is provided in Fig. 4. While prospective datasets can generate testable hypotheses and set constraints for mechanistic research (e.g. by testing whether DNAm patterns *statistically* mediate G-E effects on ADHD and whether a causal model is plausible), addressing causality will ultimately require the use of additional methods (e.g. Mendelian randomization, trio genetic designs, experimental models) and molecular studies to characterize *biological* pathways linking DNAm to ADHD (e.g. via integration with other omics data such as gene expression). If a causal role is supported, it will be equally important to determine whether modifiable pre- or postnatal environmental factors moderate the effect of DNAm on ADHD. These could be targeted in future to mitigate risk and help identify early critical periods when signals may be reversed.

The long-term goal would be to discover whether and how identified peripheral DNAm patterns relate to the brain itself—the most relevant organ for ADHD and other mental disorders. Several models have been proposed for this [98]. For example, DNAm patterns in blood or saliva may correlate with those in the brain (e.g., in response to common factors, such as prenatal exposures or genetic effects), even though changes in these tissues are not functionally linked to one another (i.e., ‘mirror’ model). Another possibility is that brain pathology causally affects peripheral processes (e.g., due to alterations in brain regions regulating neuroendocrine and hormonal function), leaving a peripheral DNAm signature (‘signature’ model). Finally, the opposite could also be true, whereby peripheral processes (e.g., systemic inflammation, circulating neurotoxins, or vascular events) affect the brain through DNAm changes (‘mechanism’ model). All three models have already received empirical support, although not specifically in the context of ADHD [100–102]. Indirect support for a *mechanism* model for ADHD comes from recent findings that systemic inflammation (cytokine levels) measured in maternal blood prenatally is related to offspring ADHD symptoms in childhood [103]. Identifying epigenetic mediators of such effects could be very powerful, with the placenta a particularly interesting primary tissue in this respect due to its importance in maternal-fetal exchange. Ultimately, establishing which of the above models applies to ADHD will directly inform translation; whereas all three models would support DNAm marks as biomarkers, only in the ‘mechanism’ model would DNAm inform etiological understanding of ADHD and potentially lead to new treatment targets.

Several strategies may be used in future to test peripheral brain associations in relation to ADHD. In humans, studies could begin by examining how the identified epigenetic signals in blood relate to individual differences in brain structure, function, and connectivity associated with ADHD. This could be achieved by leveraging recent collaborative initiatives within the emerging field of neuroimaging epigenetics, which are opening exciting opportunities to test *in vivo* associations between peripheral DNAm and the brain at different developmental stages [98] (e.g. consortia: MIND, ENIGMA-Epigenetics). As a specific example, it would be interesting to examine whether peripheral DNAm levels of *ST3GAL3* (i.e. the top ADHD GWAS hit from Demontis et al. [12] and top EWAS hit at birth from Walton et al. [64]) associate with trajectories of white matter development and integrity based on diffusor tensor imaging data, to test whether functional effects of *ST3GAL3* on myelination reported by experimental models extend to humans. At the same time, post-mortem research could be expanded in order to characterize crosstissue DNAm correspondence at different stages of life (e.g., from neonatal to adulthood), using data from large brain banks (for a review, see [104]). Ideally, available blood-brain comparison tools, which currently primarily rely on adult data (e.g. [105, 106]) would be extended in future to chart potentially dynamic changes in crosstissue concordance across development (i.e., covering time periods that are more relevant for the study of ADHD). This would allow evaluating, for example, whether cord blood DNAm shows stronger concordance with the brain compared with peripheral blood at later ages, potentially explaining why it emerges as a stronger predictor of ADHD symptoms in population-based EWASs. With regard to experimental approaches, tools for targeted epigenetic editing could be used in animal models to manipulate DNAm patterns in genes of interest identified by human studies, so as to characterize their functional effects on brain development, behavior, and ADHD risk [107]. Furthermore, experimental models could be used to complement human observational data, enabling us to further unravel genetic versus environmental influences on ADHD-related DNAm sites as well as cross-tissue correspondence. Finally, *in vitro* models may also be leveraged to investigate the effects of ADHD-associated DNAm marks at a cellular level. For this, cord blood could represent a particularly useful tissue source given that (1) DNAm levels in this tissue have been found to prospectively associate with ADHD, and (2) it contains multipotent cells that can be differentiated into neurons, thereby enabling to test whether ADHD-associated DNAm marks have functional effects on brain cell biology and behavior.

## 4 Looking to the Future: Translational Potential of Methylation-Based Applications for Personalized Medicine in ADHD

In summary, in this *Current Opinion* we have argued that (1) major needs exist for biomarker application to personalized medicine in ADHD in relation to early detection, patient stratification, and prediction of illness course and treatment response, but that (2) these efforts have been hampered by the complex and largely unknown etiology of ADHD, involving developmentally dynamic interplay between many genetic and environmental factors, likely starting in utero. Yet, we also provide a cautiously optimistic argument that (3) in response to this challenge, epigenetic processes such as DNAm have recently emerged as a molecular system of interest in the search for both ADHD biomarkers and mechanisms; (4) to date, epigenetic studies in both clinical and population-based samples have laid a solid foundation for future research, by demonstrating initial case-control findings in children and confirming early prediction of risk using cord blood; however, (5) increased consideration of developmental timing/age, tissue, and sex effects appears necessary to move this area of research forward. While the road is still long and significant technical obstacles will make progress slow, we conclude here by speculating on different ways in which epigenetic research may ultimately contribute to personalized medicine in ADHD. Realistically, given the small biological and statistical effect sizes observed in current studies for individual DNAm sites, methylation-based profiling will be likely to show greatest utility when combined with additional data (e.g., genetic, molecular, environmental, lifestyle-related) as part of a multi-modal, multi-method predictive model, such as in the case of complex disease more generally. Furthermore, as noted earlier, DNAm is only one of several epigenetic processes that interact together and likely also play a role in the development of ADHD. While we have focused on DNAm in this *Current Opinion*, as it is to date the most widely investigated and best understood epigenetic mechanism in the context of human health and disease, we believe that many of the points raised in this section on future translational potential also pertain to other epigenetic mechanisms, as research on these more challenging domains matures.

### 4.1 Methylation-Based Tools for Early Detection

First, DNAm may show utility as a predictive tool for early risk detection. In this respect, neonatal DNAm from cord blood, an easily accessible tissue, may provide a particularly promising opportunity for earlier and better detection of ADHD risk, pre-symptom manifestation. As discussed in earlier sections, this will depend on first characterizing the properties of this signal at birth (e.g., specificity and sensitivity) and establishing whether it adds sufficient predictive utility over and above known risk factors for ADHD (e.g., amplifying the utility of early inflammation markers in a complex biomarker model for ADHD risk antenatally).

A critical question will be whether the benefits of neonatal methylation-based screening for ADHD, somewhat like genetic screening, will outweigh potential risks and ethical concerns. On the one hand, population-level neonatal screening (e.g., via heel prick-derived blood) is already routinely performed in many countries to detect a range of diseases (e.g., metabolic and adrenal disorders, sickle cell anemia, and others). Many of these cannot yet be cured, but early detection and intervention (e.g., via dietary advice or medication) has been effective in preventing or reducing risk for serious impairments. On the other hand, neonatal screening comes with a number of well-known caveats, including the risk of false positives, the fact that non-specific biological risk markers may never materialize into actual impairment, the possible introduction of unnecessary interventions, the potential for increased stigma unless screening is appropriately communicated and managed, as well as logistical and financial challenges related to the implementation of large-scale screening programs.

These concerns are amplified for ADHD because screening would detect *risk* for a complex phenotype with unclear etiology, as opposed to high probability identification of Mendelian disorders or other disorders with known etiology. Furthermore, we still do not have an effective early intervention to *prevent* ADHD development due to the absence of known mechanisms, which raises questions about how actionable a positive screening result would be. It remains unknown to what extent nutritional, medical, or behavioral (e.g., parenting skills) interventions in the first months of life may be able to alter ADHD risk trajectory sufficiently to justify screening-related risks. Perhaps, early nutritional intervention could rescue inflammation-related risk [108], whereas early intervention around parental attunement and stress management with a difficult-to-manage child could enable better parent/child relations [109] and protective factors such as longer breast-feeding [110] that overcome other risks. To date, behavioral and other interventions for ADHD in the late preschool years (e.g., ages 3-5 years) have had only limited success [, 111], suggesting that even earlier intervention would be preferred if appropriate mechanisms and methods were identified. Taken together, these concerns mean that even if we reach the stage of developing effective methylation-based neonatal screening tools, careful assessment of costs, risks, ethical implications, social acceptability, and actionability will be essential before such a tool can be successfully implemented. Nonetheless, the long-term potential should not be ignored and commends continued study in this direction.

### 4.2 Methylation-Based Tools for Patient Stratification and Subtyping

A second area where methylation-based tools could benefit ADHD research and clinical practice is patient stratification and subtyping for treatment planning. Genomic prediction for medication assignment is still in the early stages, and biomarkers that can predict ADHD course are extremely limited, as we noted earlier. DNAm profiling could be used in conjunction with other information (e.g., phenotype profile, polygenic risk scores, demographic variables, exposure risks, and lifestyle factors, as well as neurodevelopmental and early behavioral markers) to predict factors such as disorder severity and chronicity as well as risk for comorbidity and the emergence of secondary health conditions. Furthermore, the use of person-centered approaches may enable the identification of subgroups of individuals with ADHD who differ in their epigenetic profiles, potentially in response to different risk factors or underlying mechanisms, helping to explain individual etiology. Although we are skeptical about the potential utility of DNAm for the diagnosis of ADHD rather than its more likely potential in risk prediction and stratification, it is notable that tools relying on ‘episignatures’ from peripheral blood have already been developed for the diagnosis of a wide range of *Mendelian* neurodevelopmental disorders, demonstrating utility for brain-based disorders [37]. To what extent epigenetic patterns associated with ADHD may also reflect (common and/or rare) genetic mutations, and whether this information could one day be used to improve diagnostic accuracy, is an important topic for future research.

### 4.3 Methylation-Based Tools for Treatment Response

Finally, DNAm profiling may show utility in future as a decision support tool to help predict and monitor therapeutic responses to different types of treatments. For example, studies in animals and humans have found that peripheral DNAm patterns associate with commonly administered psychoactive medications, including mood stabilizers and antipsychotic and antidepressant drugs [112]. Pharmacogenomics in psychiatry is a rapidly developing field, although at the level of FDA recognition its utility for ADHD remains extremely limited [113]. In future, larger, multi-site collaborative projects will be needed to identify epigenome-wide marks associated with treatment response (which may be used to develop aggregate polyepigenetic scores) and to more stringently account for other potential factors that may account for observed associations (e.g., environmental factors, polymedication use, differences in symptom severity, etc.). Once robust marks are identified, these will also need to be validated using controlled clinical trials. Of note, the temporally dynamic and environmentally sensitive nature of DNAm may be particularly useful as a leading indicator of symptom status and change, or it may add problematic complexity to the development of clinically informative methylation-based tools. Ultimately, DNAm marks associated with treatment response may also shed light on the pathophysiology of ADHD, enabling the identification of new potential drug targets.

## 5 Conclusion

The field of epigenetics and ADHD is still in its infancy, and concrete translational applications are a distant goal. Yet, there is much to be (cautiously) optimistic about. The field is at an exciting stage as it moves past initial association studies in pursuit of new discoveries in the areas of biomarker identification, beginning in early life and across development. Indeed, initial reports of replicated case-control differences and epigenetic timing effects on ADHD symptoms signals the promise now emerging in this new field, while the recent establishment of multi-site, international collaborations to pool data and advance knowledge on the epigenetics of ADHD augers well for continued growth in the coming years. Ultimately, the potential of DNAm for biomarker and mechanism discovery on the one hand, as well as its link to exposure and genetic influences on the other, continues to make this a compelling candidate molecular tool for future personalized medicine in ADHD.

## Figures and Tables

**Table 1:** Overview of epigenome-wide association studies of ADHD to date

References	Study characteristics					DNA methylation		Phenotype definition (instrument)	Findings
	Design (*A* time points DNAm)	Single or multicohort	Developmental period (age of outcome assessment)	Sex (% males)	Sample	Array	Tissue		
Clinical and high-risk samples									
Wilmot et al. [54]	Cross-sectional (1)	Single (split into discovery and confirmation set)	Childhood (7-12 years; mean = 9 years)	Males (100%)	*Discovery set: N* = 92 (43 with ADHD vs. 42 healthy controls); *Confirmation set: N* = 20 (\Owith ADHD vs. 10 healthy controls). Predominantly European ancestry	450k	Saliva	Clinically established ADHD cases vs. controls (KSAD-S-E)	Genome-wide significance not tested. DNAm differences between cases and controls in two genes. *VIPR2* and *MYT1L*, met pre-defined criteria for follow-up analyses for confirmation testing. DNAm differences in *VIPR2* were also observed in the confirmation set and via bisulfite sequencing. Enriched pathways related to inflammatory processes. fatty acid oxidation, as well as modulation of dopaminergic and cholinergic neurotransmission
Chen et al. [55]	Cross-sectional, twin discordant analyses (1)	Single	Childhood (mean = 11 years)	Males and females (86%)	*N* = 14 pairs of monozygotic twins discordant for ADHD. European ancestry	450k	Peripheral blood (whole)	Clinically established ADHD in discordant twins (DICA)	Significant enrichment of epigenetic differences in genes expressed by brain regions associated with ADHD discordance (e.g., striatum and cerebellum-expressed genes). Some evidence of differential *VIPR2* methylation. Enriched biological pathways included GABA, dopamine and serotonin neurotransmission
Mooney et al. [56]	Cross-sectional (1)	Single	Childhood (7-12 years; mean = 10 years)	Males and females (*72%* in cases; 52% in controls)	*N* = 542 (391 with clinically established ADHD vs. 213 nonpsychiatric controls). Predominantly European ancestry	EPIC	Saliva	Clinically established ADHD cases vs. controls (KSADS-E)	No genome-wide significant associations with case-control status, although results support previously identified differences in *VIPR* methylation. One genome-wide significant DNAm site associated with polygenic risk for ADHD (cgl5472673 annotated to promoter of *GART* and *SON*)
Meijer et al. [57]	Cross-sectional (1)	Single	Adolescence (12-23 years)	Males and females (73%)	*N* = 72 (35 with persistent ADHD. 18 with remittent ADHD, and 19 healthy controls). Selected subsample from the NeuroIM-AGE study. European ancestry	EPIC	Peripheral blood (whole)	ADHD cases identified by combining multiple instruments (KSADS. PACS). based on DSM-IV criteria. Further distinction between remittent vs. persistent ADHD cases	No genome-wide significant differences identified between ADHD cases vs. controls. Differentially methylated regions in *APOB* and *LPAR5*, two genes involved in cholesterol signaling, were observed between individuals with persistent vs. remittent ADHD. These differences were associated with *cis* mQTLs. suggesting genetic influences
Rovira et al. [58]	Cross-sectional (1)	Single	Adulthood (meancases = 32 years; meancontrols = 37years)	Males and females (56% in cases; 45% in controls)	*N* = 203 (103 individuals with ADHD vs. 100 healthy controls). European ancestry	EPIC	Peripheral blood (mononuclear cells)	Clinically established ADHD cases vs. controls (assessed primarily via CAADID and supplemented with multiple other instruments)	At a genome-wide level, one DNAm site (cgO7143296, annotated to *PCNXL3*) and four regions (mapping to *DENND2D*, *PWWP2B*, *AKO94674* and *UBASH3A*) were found to be differentially methylated between cases and controls. Findings were not explained by smoking or polygenic risk for ADHD, although epigenetic signatures did overlap with those previously identified for smoking-related traits and behaviors
Population-based samples									
Walton et al. [64]	Prospective and cross-sectional (2)	Single	Childhood to adolescence (7-15 years)	Males and females (50%)	*N* prospective analyses = 817 (40 high ADHD trajectory vs. 777 low trajectory); *N* cross-sectional analyses = 892 (50 high trajectory vs. 842 low trajectory). Predominantly European ancestry	450k	Cord and peripheral blood (whole)	Longitudinal trajectories of ADHD symptoms (DAWBA) based on assessments at age 7, 10, 13, and 15 years	In prospective analyses. DNAm patterns at birth were found to differentiate between ADHD symptom trajectories (13 genome-wide corrected DNAm sites). These sites mapped to genes implicated in perixosomal processes (e.g. fatty acid metabolism). neural tube development, myelination, and ADHD. One of these genes. *ST3GAL3*. was also independently identified as a top hit for ADHD based on GWAS. In contrast, no associations were identified in cross-sectional analyses between DNAm measured in childhood and ADHD trajectories, suggesting time-specific epigenetic effects
Neumann et al. [72]	Prospective and cross-sectional (2)	Multi (metaanalysis)	Childhood (prospective analyses: 4-15 years; cross-sectional analyses: 7-11 years)	Males and females (50)	Pooled *N*. prospective analyses = 2477 (5 cohorts); Pooled *N*, cross-sectional analyses = 2374 (9 cohorts, of which 3 were also included in the prospective analyses). Predominantly European ancestry	450k	Cord and peripheral blood (whole)	ADHD symptoms analyzed dimensionally (DAWBA. CBCL. Conners. or BASC. depending on the cohort)	In prospective analyses. DNAm patterns at birth were found to associate with ADHD symptom severity across nine DNAm sites, after genome-wide correction. These sites mapped to genes such as *ERC2* and *CREB5* were previously implicated in neurotransmitter release, neurite outgrowth, and ADHD diagnosis. In contrast, no genomewide significant associations were identified in cross-sectional analyses (i.e.. when DNAm and ADHD symptoms were measured at the same time point in childhood)
van Dongen et al. [76]	Cross-sectional (1)	Multi (metaanalysis)	Adulthood (mean age ranging between 18 and 38 years across cohorts)	Males and females (31–50% depending on the cohort)	Pooled *N* = 4689 (3 cohorts)	450k	Peripheral blood (whole)	ADHD symptoms analyzed dimensionally (CAARS or structured interviews, depending on the cohort)	No differentially methylated DNAm sites or regions were identified in the pooled meta-analysis after genomewide correction. Of the non-over-lapping differentially methylated regions identified in individual cohorts, the majority were associated with mQTLs and showed moderate brain–blood correlations. Several of these mapped to the major histocompatibility complex implicated in immune function

*ADHD* attention-deficit/hyperactivity disorder, *GWAS* genome-wide association study, *DNAm* DNA methylation, *DSM-IV* Diagnostic and Statistical Manual of Mental Disorders, 4th Edition, *KSADS* Kiddie Schedule for Affective Disorders and Schizophrenia. *KSADS-E* Kiddie Schedule for Affective Disorders and Schizophrenia - Epidemiological version, *DICA* Diagnostic Interview for Children and Adolescents, *PACS* Physical Appearance Comparison Scale, *CAADID* Conners’ Adult ADHD Diagnostic Interview for DSM-IV, *DAWBA* Development and Well-Being Assessment, *CBCL* Child Behavior Checklist, *BASC* Behavior Assessment System for Children, *CAARS* Conners’ Adult ADHD Rating Scales, *mQTL* methylation quantitative trait loci

## Data Availability

Data sharing not applicable to this article as no datasets were generated or analysed during the current study.
